# Extracellular Microvesicles Released From Brain Endothelial Cells are Detected in Animal Models Of HIV-1 Signifying Unresolved Inflammation

**DOI:** 10.1007/s11481-021-10008-5

**Published:** 2021-08-26

**Authors:** Servio H. Ramirez, Tetyana P. Buzhdygan, Jonathan F. Hale, Liang Cheng, Guangming Li, Bryson Hoover-Hankerson, Roshanak Razmpour, Uma Sriram, Lishan Su, Raghava Potula, Allison M. Andrews

**Affiliations:** 1 Department of Pathology &, Laboratory Medicine Lewis Katz School of Medicine at Temple University, 3500 N Broad St, PA 19140 Philadelphia, USA; 2 The Center for Substance Abuse Research Lewis Katz School of Medicine at Temple University, 3500 N Broad St, PA 19140 Philadelphia, USA; 3 The Shriners Hospitals Pediatric Research Center, Philadelphia, PA 19140 USA; 4 Division of Virology, Pathogenesis and Cancer, Institute of Human Virology, Departments of Pharmacology, University of Maryland School of Medicine, Baltimore, MD 21201 USA; 5 Lineberger Comprehensive Cancer Center, Department of Microbiology and Immunology, The University of North Carolina At Chapel Hill, Chapel Hill, NC 27599 USA; 6 Frontier Science Center for Immunology and Metabolism, Medical Research Institute, Wuhan University, Wuhan, 430071 China

**Keywords:** Blood–brain barrier, HIV, Microvesicles, Extracellular vesicles, Neuroinflammation, Biomarkers, NeuroHIV

## Abstract

Treatment of HIV-infected patients with antiretroviral therapy (ART) has effectively suppressed viral replication; however, the central nervous system is still a major target and reservoir of the virus leading to the possible development of HIV-associated neurocognitive disorders (HAND). Furthermore, a hallmark feature of HAND is the disruption of the blood–brain barrier that leads to loss of tight junction protein (TJP) complexes. Extracellular vesicles (EVs), released by every cell type in the body, occur in greater quantities in response to cellular activation or injury. We have found that inflammatory insults activate brain endothelial cells (EC) and induce the release of EVs containing TJPs such as Occludin. We thus hypothesized that HIV infection and unresolved neuroinflammation will result in the release of brain-EC derived EVs. Herein, our results show elevated levels of brain-EC EVs in a humanized mouse model of HIV infection. Furthermore, while ART reduced brain-EC EVs, it was unable to completely resolve increased vesicles detectable in the blood. In addition to inflammatory insults, HIV-1 viral proteins (Tat and gp120) increased the release of Occludin + vesicles from human brain microvasculature ECs. This increase in vesicle release could be prevented by knock-down of the small GTPase ARF6. ARF6 has been shown to regulate EV biogenesis in other cell types, and we provide further evidence for the involvement of ARF6 in brain EC derived EVs. Overall, this study offers insight into the process of brain vascular remodeling (via EVs) in the setting of neuroinflammation and thus provides possibilities for biomarker monitoring and targeting of ARF6.

**Graphical abstract:**

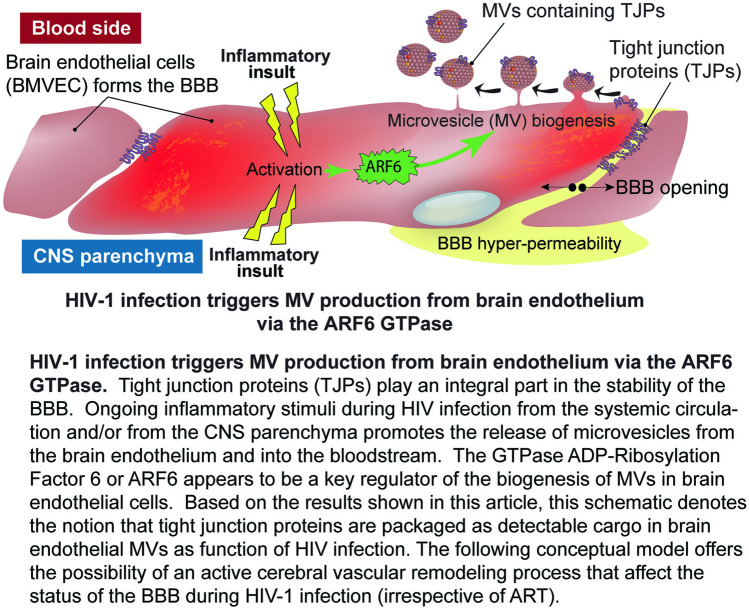

## Introduction

Although the use of antiretroviral therapy has reduced the prevalence of HIV-associated dementia (HAD), the overall rates of HIV-associated neurocognitive disorders (HAND) remains unchanged (Saylor et al. 2016). Underlying these disorders is the notion that immune activation, neuroinflammation and disruption of the blood–brain barrier (BBB) play a central role in the development of HAND (Chaganti et al. 2019). The BBB is comprised of endothelial cells (EC) that form interlocking tight junction protein complexes with neighboring ECs that restricts the entry of blood solutes into the CNS. It is known that the consequences of HIV infection, including inflammation and HIV viral proteins, induces changes to the junction proteins leading to decreased barrier function. 

Extracellular vesicles (EVs), which include exosomes, microparticles/microvesicles (MVs), and apoptotic bodies, are small membrane bound compartments that range from 50-1000 nm in diameter (Muralidharan-Chari et al. 2010). EVs are released or secreted in response to stimuli, injury, or activation from a number of cell types (platelets, immune cells and ECs) (Boulanger et al. 2006). Due to the faster release kinetics of MVs, and their level of cellular surface markers to determine origin, our studies presented in this report focuses on MVs rather than the other subcategories of EVs. MVs are isolated by differential centrifugation and are detectable by flow cytometry, mass spectrometry, electron or atomic force microscopy and capture-based assays such as ELISA. While the exact function of MVs remains unclear, studies have shown that they can contribute to pathology (Candelario and Steindler 2014; Anderson et al. 2010) or serve as a form of intercellular communication (Pegtel et al. 2014). HIV-1 infection or viroproteins triggers the production of MVs from various cell types, including but not limited to monocytes (Weber et al. 2020; Kadiu et al. 2012), microglia (Wu et al. 2015), platelets (Poveda et al. 2020), and peripheral ECs (Hijmans et al. 2019a). Furthermore, there is great interest in the possibility that MVs (and the other EV forms) could be used as biosignatures for monitoring HIV related pathogenesis (Weber et al. 2020; Falasca et al. 2021; Bazie et al. 2021; Pulliam et al. 2019). Although studies have shown that HIV-1 promotes MV production from distinct cell populations, limited information is available regarding the cells that form the BBB.

A key defining aspect that separates the different subtypes of EVs is the mechanism of formation. MVs form from a budding of the plasma membrane and commonly includes the influence of small GTPases (Clancy et al. 2021). In particular, ARF6 has been shown to mediate the release of membrane-derived MVs by regulation of actomyosin contraction at the necks of the MVs (Muralidharan-Chari et al. 2009; Sedgwick et al. 2015). We have previously reported that ARF6 activation was linked to vesicle production after mechanical injury (Andrews et al. 2016), however, whether ARF6 is a master regulator of MV release from human brain microvascular endothelial cells (hBMVECs) in response to multiple insults remains unknown.

We have previously published that after cytokine or mechanical insult, hBMVECs release increased levels of MVs that also contain tight junction proteins as part of their surface cargo (Andrews et al. 2016; Ramirez et al. 2018). Thus, studies herein were aimed at determining whether in the context of HIV-1 infection brain ECs respond similarly in elevating the production of MVs and whether such release correlates with changes to BBB tightness. In addition, given the importance of ARF6 in EV biogenesis, we sought to strengthen the evidence of the involvement of ARF6 in the regulation of MV production from BMVECs.

## Materials & Methods

### Reagents

Rat-tail collagen type I and endothelial cell growth supplement (ECGS) were purchased from BD Biosciences (Franklin Lakes, NJ). Heparin and lipopolysaccharide (sigma), DMEM/Ham’s F12 media, Fetal bovine serum (FBS) (Thermo Fisher Scientific, Waltham, MA). Recombinant human TNF-α was purchased from R&D Systems (Minneapolis, MN, USA). Gp120 HIV IIIB and Tat IIIB were purchased from ImmunoDX (Woburn, MA, USA). pcDNA3-EGFP. (Plasmid #13,031, deposited by Doug Golenbock) acquired form Addgene (Watertown, MA, USA) was used in control transfections. ARF6 constructs (pARF6-T27N-CFP #11,386, pARF6-YFP #11,389, pARF6-Q67L-CFP #11,387, and CFP) were a gift from Joel Swanson via Addgene. Bacterial stabs from Addgene were streaked on standard Luria broth agar plates containing kanamycin. Single colonies were isolated and suspension cultures grown for utilization with plasmid isolation Maxi kits (Qiagen).

### Endothelial Cell Culture

All experiments were performed with primary (hBMVEC) using a minimum of 3 donors from adult resected tissue or human fetal tissue sources. Primary human (adult) hBMVECs were provided by Michael Bernas and Dr. Marlys Witte (University of Arizona, Tucson). hBMVECs were isolated from vessels from the brain resection path of patients (showing no abnormalities) undergoing surgery for the treatment of intractable epilepsy, as previously described (Bernas et al. 2010).

Primary (fetal) hBMVECs were isolated from healthy tissue from fetal brains (112–154 days) obtained from the Birth Defects Research Laboratory (University of Washington, Seattle, WA) in full compliance with Temple University (Philadelphia, PA) and the National Institutes of Health’s (NIH) ethical guidelines. Isolation has been previously described (Andrews et al. 2018). In short, brain tissue was washed in 2–3 × in Hank’s Balanced Salt Solution (HBSS) and pelleted at 1000 rpm for 5 min, the tissue (1–5 cm^3^) was then digested in 0.5% collagenase in PBS at 37 °C for 40 min. Following digestion, the tissue suspension was washed with HBSS, centrifuged at 1000 rpm for 5 min and the supernatant discarded. The pellet was resuspended in 12 mL sterile 17.5% dextran (MW 86.000, MP Biomedicals, LLC) in HBSS. Vessels were pelleted by centrifugation at 4400 × g for 15 min. Vessels were isolated from the wall of the centrifuge tube and passed through sequential filters (100 μm and 40 μm, Falcon). Vessels caught in the 40 μm filter were then plated onto human fibronectin (Thermo Fisher Scientific) coated 6-well dishes (Corning). fBMVECs were sorted as previously described (Andrews et al. 2018), to ensure endothelial purity.

For experimental conditions, cells were cultured in our “endothelial growth media” until confluency (Bernas et al. 2010). The endothelial growth media consisted of Dulbecco's modified Eagle's medium/F12 media supplemented with 10% heat-inactivated fetal bovine serum (FBS; Thermo Fisher Scientific), endothelial cell growth supplement (ECGS) (BD Bioscience), heparin (1 mg/mL; Sigma/Aldrich Co, Ltd), amphotericin B (2.5 μg/mL; Thermo Fisher Scientific), penicillin (100 U/mL; Thermo Fisher Scientific), and streptomycin (100 μg/mL; Thermo Fisher Scientific). Upon confluency, the media was exchanged for media without added growth factors.

For stimulation and collection of MVs: Cells were washed with PBS and treated with DMEM/F12 with EV-depleted FBS (Systems Biosciences, SBI). Experiments were performed as paired treatments, first obtaining a baseline reading in media without insult, followed by the treatment with insult dissolved in the media for the same duration of time (6 h or 24 h). Insults included 100 ng/mL TNF, 100 ng/mL LPS, 100 ng/mL gp120 and 100 ng/mL Tat. Media was collected and centrifuged at 2000 g for 20 min. Supernate was snap frozen in liquid N2 and stored at –80C until use.

### Humanized Mice

Cohort #1: NOD.Cg-*Prkdc* ^scid^ IL2rg ^tm1Wjl^ /SzJ mice (Nod-SCID-common gamma chain knockout [NSG] engrafted with human CD34 + hematopoietic stem cells were purchased from The Jackson Laboratory (Sacramento, CA USA). Mice were maintained in microisolator cages in appropriate sterile conditions, which include autoclaved cages, bedding, nestlets, and igloos along with irradiated food and filtered water. Reconstitution was quantified from mouse peripheral blood collected from the submandibular vein by evaluation of human CD45^+^ using flow cytometry (data not shown). Mice with 25% or higher human CD45^+^ engraftment were included in the study. All animal experiments were approved by the Temple University Institutional Animal Care and Use Committee and conducted in accordance with the Temple University guidelines. Temple’s guidelines are based on the National Institutes of Health (NIH) guide for care and use of laboratory animals and with the ARRIVE (Animal Research: Reporting In Vivo Experiments) guidelines. Humanized NSG (NSG-hu) mice were infected with the HIV-1_ADA_ strain at a multiplicity of infection (MOI) of 0.1 infectious particles in a volume of 50 µl per mouse. In this cohort (of 13 mice), blood was collected and plasma was isolated before HIV-1 infection (time 0). Infected animals selected for this study are shown in the graph. Animals excluded from the study include engraftment failure or for other welfare conditions. Plasma viral load (copies/ml) was measured by RealStar HIV RT-qPCR kit 1.0 (Altona Diagnostics, Hamburg, Germany) as per manufacturer’s instructions.

Construction of humanized mice for cohort #2: Human fetal liver tissues were obtained from elective or medically indicated termination of pregnancy through a non-profit intermediary working with outpatient clinics (Advanced Bioscience Resources). Approval for animal work was obtained from University of North Carolina (UNC) Institutional Animal Care and Use Committee (IACUC, ID 16–073). We constructed CD34^+^ hematopoietic stem cells (HSC) transplanted humanized NSG (NOD-scid IL2rgnull) mice (hu-NSG) through intra-liver injection of 2 × 10^5^ (Candelario and Steindler 2014) purified HSC individually (Li et al. 2014). Human immune cell engraftment was detected by flow cytometry 12 weeks after transplantation. In the cohort (of 12 mice), 8 mice were infected and 4 mice were mock control. 4 of the infected mice received ART starting from 4wpi. All mice were terminated at 12wpi.

For infection, HIV-1_JR-CSF_ was generated by transfection of 293 T cells using calcium phosphate method. Plasmids were obtained from NIH AIDS Reagent Program. Virus titer was measured by either p24 ELISA (Cell Biolabs) and MAGI-CCR5 (Pirounaki et al. 2000; Kimpton and Emerman 1992). Animals were infected with HIV-1_JR-CSF_ at a dose of 10 ng p24/mouse, through retro-orbital injection.

### Combination Antiretroviral Therapy (cART)

Individual tablets of TRUVADA (tenofovir/emtricitabine; Gilead Sciences) or raltegravir (Merck) were crushed into fine powder and manufactured as 5BXL by TestDiet based as previously published (Halper-Stromberg et al. 2014; Cheng et al. 2018). HIV-1 RNA was extracted from plasma (Viral RNA Mini Kit, Qiagen). RNA was reverse transcribed and quantitatively detected by real-time PCR using TaqMan® Fast Virus 1-Step PCR kit (ThermoFisher Scientific) and QuantStudio 6 Flex PCR system (Applied Biosystems) (detection limit ≈400 copies/ml) (Li et al. 2014, 2017).

### Isolation and Flow Cytometry of EVs

MVs were isolated using differential centrifugation. In short, media or plasma was first centrifuged at 2000 × g for 20 min. The supernate was then centrifuged at 20,000 × g for 40 min using a Beckman Coulter Benchtop Ultracentrifuge. Pelleted MVs were washed in sterile filtered (0.22um) Phosphate-buffered saline (PBS) and respun. MVs were then resuspended in 100 µL of flow cytometry buffer with primary antibody Occludin (Alexa488, abcam for mouse or Invitrogen for human) with or without primary antibody for CD31 (BV421, biolegend) for 30 min. Sample was washed with PBS and respun. Samples were then resuspended in 200 uL and data was collected over a fixed time (2 min) at a constant flow rate for two replicates of the same sample in a FACS BD Canto II flow cytometer (BD Biosciences) and analyzed with FlowJo software (FlowJo LLC, a subsidiary of Becton Dickinson, Ashland, OR, USA). The two replicates for each sample were then averaged. Experiments were performed 7–14 times with multiple biological replicates of each of multiple donors.

### Trans-Endothelial Electrical Resistance (TEER)

hBMVECs were plated (20,000/well) on collagen I coated 96-well electrode arrays (96W20idf, Applied Biophysics, Troy, NY, USA) and grown until confluent. Trans-endothelial electrical resistance (TEER) was measured at 2 kHz every 600 s using the Electrical Cell-Substrate Impedance Sensing (ECIS) system (Applied Biophysics, Troy, NY, USA) as described previously (Buzhdygan et al. 2021). Cells were grown until a constant baseline resistance was obtained and then media was replaced with lack of growth factors. Cells were treated with 100 ng/mL of TNF, LPS, gp120 or Tat, dissolved in media (without growth factors) and resistance was measured for 24 h. Results represent 3–5 replicates from each donor.

### ARF6 Activation Assay

ARF6 activation was determined using the ARF activation assay (Cell Biolabs, inc.). GGA3 PBD Agarose beads were used to selectively isolate the active form of ARF from cell lysates as described by the manufacturer’s instructions. In short, cells were grown to 80–90% confluency and then stimulated for 5 or 15 min. Media was removed and cells were washed, lysed, cleared by centrifugation (10 min 14,000 g at 4C) and then the supernate was snap frozen and stored at –80C. GGA3 PDB Agarose beads were added to the samples and incubated for 1 h at 4C. Beads were then pelleted by centrifugation at 14,000 g for 10 s, and washed 3 times before loading onto a 4–20% Mini-Protean TGX gel (Biorad). Proteins were transferred onto nitrocellulose by semi-dry transfer with the Trans-blot Turbo (Biorad). Membranes were blocked with SuperBlock (Thermo Fisher) and all primary (1:1000) incubated overnight and HRP secondary (1:10,000) antibodies incubated for 1 h were dissolved in SuperBlock (Thermo Fisher). Bound antibodies were exposed to Supersignal West Pico chemiluminescent substrate (Thermo Fisher) and then visualized by the gel documentation system, G:Box Chemi HR16 (Syngene, Frederick, MD).

### ARF6 Knockdown and ARF6 Plasmid Transfection

hBMVECs were transfected using the Neon Transfection System (Fisher) following the manufacturers protocol. Cells was electroporated using one pulse of 1,100 V for 30 ms. The use of the Neon transfection system hBMVECs provided 30%–60% transfection efficiency. For knockdown experiments, 50 nM of ARF6 smart pool siRNA or ON-Target plus Non-Targeting siRNA (Dharmacon).

After 72 h, cells were treated with EV-free media without growth factors for 6 h followed by treatment with insult for 6 h. For transfected cells with ARF6 plasmids, cells were visualized at 72 h after transfection using an A1R Nikon Confocal. Images were taken with image acquisition and analysis software, NIS Elements AR (Nikon).

### Statistical Analysis

The experiments were independently performed multiple times (at least three times for all the data shown) to allow statistical analyses. Within each individual experimental set, every condition was evaluated in a minimum of three replicates. The data collected were analyzed using Prism v6.0c software (Graphpad Holdings, LLC, San Diego, CA). Multiple group comparisons were performed by two-way analysis of variance (ANOVA) with post-hoc analysis or students t-test where indicated. All the results are expressed as the mean ± SEM with differences considered significant at *p* < 0.05.

## Results

### Animals infected with HIV-1 generate higher number of EVs Containing Brain Endothelial Derived Microvesicle EVs

We have previously reported that neuroinflammation in vivo (Andrews et al. 2016) and primary hBMVECs exposed to HIV viral proteins (Ramirez et al. 2018) produce vesicles containing tight junction proteins. Since MVs contain cell-specific protein cargo, identification of the cell of origin for the MVs can be determined. Thus, we utilized a dual-protein marker detection approach to interrogate serological MVs for their cell origin. To determine the likelihood that the MVs are of cerebral vascular origin, both CD31 (an endothelial marker) and Occludin (a highly enriched tetraspanin expressed in the cerebral endothelium) were utilized. MV production can signify remodeling, activation and pro-inflammatory response in ECs. To evaluate whether MVs released from the brain vasculature occurs during HIV-1 infection analysis was performed in relevant animal models.

MVs, were analyzed by flow cytometry from samples collected from two cohorts of animal models of HIV-1. Viral load determinations in plasma confirmed infection in hu-NSG mice and ranged from 10^3^ to 10^5^ viral copies/ml.

**Fig. 1:**
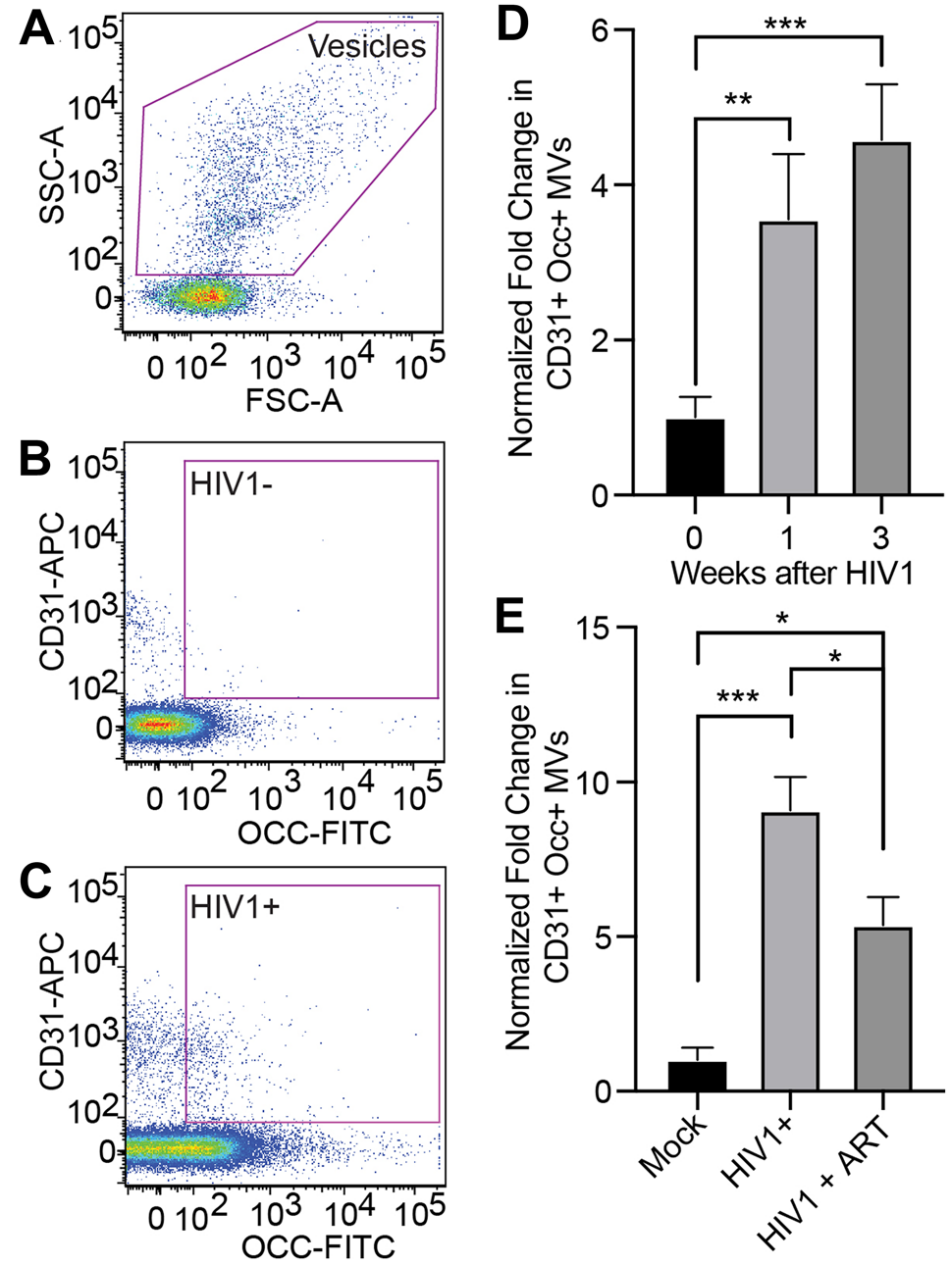
HIV Infection increases brain endothelial microvesicle release in vivo. Microvesicles (MVs) were isolated from the plasma of hu-NSG animals by ultracentrifugation. MVs labeled with CD31-APC and Occludin-FITC. **A**. MVs were first gated on vesicles. Representative dot plot of double positive vesicles (Red box) isolated from HIV negative (**B**) and HIV positive (**C**) animals. **D**. Vesicles were isolated from cohort #1 prior to HIV infection (timepoint 0) and at 1- and 3-weeks post-HIV infection. HIV infection statistically increased double positive MVs (CD31 + Occ +) counts (one-way ANOVA **p < 0.01, ***p < 0.001, n = 3–9 each condition). **E**. Vesicles were isolated from Cohort #2 of hu-NSG mice. Groups included mock infected, infected with HIV (12 wpi), or infected with HIV and treated antiretroviral therapy (ART, from 4–12 weeks). HIV infection increased double positive MV counts to ~ ninefold of mock infected animals. Double positive MV counts were also increased in HIV infected animals on ART but it was significantly reduced compared to HIV infection alone (one-way ANOVA *p < 0.05, ***p < 0.001, n = 3–4 each group)

First using differential ultra-centrifugation, MVs were isolated and purified from the blood plasma of uninfected or HIV-1_ADA_ infected NOD-scid mice (homozygous for a deletion of the IL-2R γ-chain) reconstituted with engrafted human tissues and CD34 + HSPCs. As shown in Fig. 1 vesicles were gated for populations that corresponded to double positive CD31-APC and Occludin-FITC in HIV- (Fig. 1B) and HIV + samples (Fig. 1C). In the first cohort, samples were analyzed before infection and at 1 and 3 weeks post-infection (Fig. 1D). HIV infection produced a 3.55 fold (± 1.46 SD) and 4.58 fold (± 1.25 SD) increase in double positive MVs at 1- an 3- weeks respectively. To further confirm these results and to determine whether ART may affect MV release during chronic infection and under combination ART, samples were analyzed in a second cohort of animals (Fig. 1E). Samples were collected at 12-weeks post-HIV-1_JR-CSF_ infection with ART-treated mice from 4–12 weeks post-infection. Animals infected with HIV had a 9.1 fold (± 1.88 SD) increase in double positive MVs (Fig. 1E). Infected animals that were treated with to control viral load also reduced double positive MVs. HIV-1 and treated with ART animals still had a 5.35 fold increase in double positive MVs compared to non-infected animals, but this was significantly reduced compared to HIV infected mice without ART (Fig. 1E). Overall, this supports the notion that HIV infection, which leads to well documented neuroinflammation, induces cerebral endothelial vascular changes that could be identified by brain EC MVs in the systemic circulation. Furthermore, while ART can reduce viral load and brain-EC MVs, it is unable to completely reverse persistent brain endothelial remodeling.

### Decrease in Barrier Integrity Corresponds with EV Production to HIV-1 Viral Proteins

It has been established that various insults to the cerebral vasculature result in the loss of tight junctions and a decrease in of BBB integrity. This loss of barrier integrity can be monitored using trans-endothelial electrical resistance (TEER) in which a decrease in resistance reflects increased permeability and endothelial dysfunction. hBMVECs were grown on 96-well coated electrodes and treated with 100 ng/ml of TNF-α, LPS, gp120 or Tat for 24 h. All insults rapidly decreased the tightness of the monolayer within the first 6 h. In the case of TNF-α and LPS, the decline in resistance continued until approximately 20 h while gp120 and Tat leveled off around 10 h and then began to recover by 24 h.

**Fig. 2:**
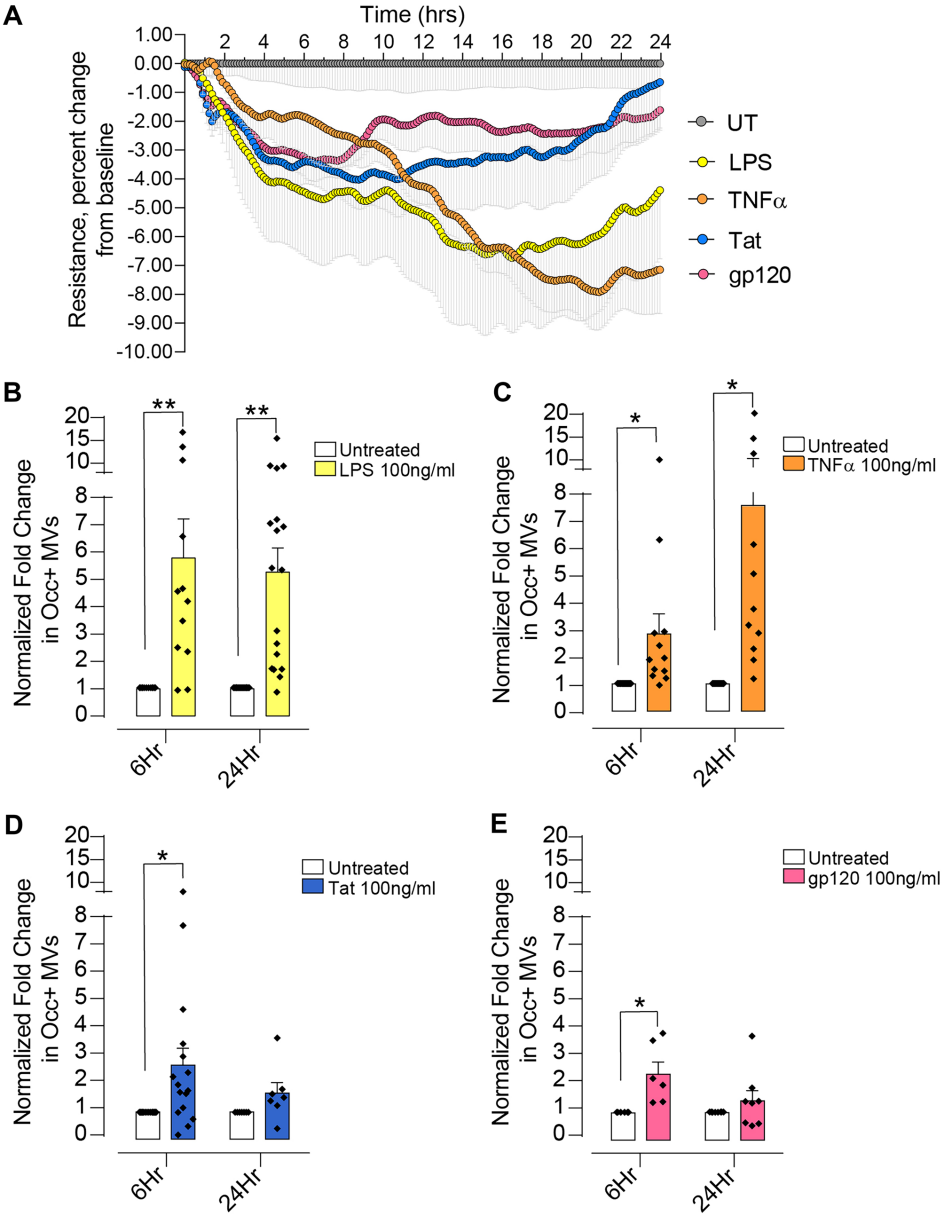
HIV viroproteins and inflammatory insults disrupt the primary human brain microvasculature endothelial barrier and corresponds to an increase in microvesicles containing the tight junction protein occludin. A. hBMVECs were grown on 96-well electrodes and electrical resistance was measured using the Z-Theta (Applied Biophysics). Cells were stimulated with 100 ng/mL of HIV-1 proteins Tat and gp120 (**A**) or 100 ng/mL of inflammatory insults TNF-α and LPS (**B**). All insults resulted in a reduction in electrical resistance equating to an increase in permeability in the endothelial monolayer. Similarly, hBMVECs grown in 100 mm dishes were treated with 100 ng/ml of LPS (**B**) TNF-α (**C**), Tat (**D**) and gp120 (**E**) for 6 h and 24 h. Media was harvested, MVs were isolated by ultracentrifugation, labeled with Occludin-FITC and analyzed by flow cytometry. Data was normalized by the basal production over the same treatment time from the same cells (paired study). (*n* = 7–18 all conditions, students t-test, *p < 0.5 **p < 0.01)

To determine the relationship between changes in barrier tightness and MV production, cells were treated with 100 ng/ml of TNF-α, LPS, gp120 or Tat. Media was collected at 6 h and 24 h and vesicles were isolated, labeled and analyzed by flow cytometry. LPS, used here as pro-inflammatory mediator in endothelial cells resulted in a 5.79 fold (± 4.93 SD) increase in occludin + MVs at 6 h and which remained elevated at 5.27 (± 3.75 SD) fold increase after 24 h (Fig. 2C). TNF-α increased occludin + MVs to 2.82 fold (± 2.52 SD) at 6 h and further increased to 7.5 fold (± 8.08 SD). HIV-1 viral proteins elicited an increase in occludin + MVs, although reduced compared to the inflammatory insults. After Tat treatment, MVs increased by 2.70 (± 2.47 SD) fold increase at 6 h and a 1.68 fold (± 1.00) fold increase at 24 h. Similarly, gp120 increased MVs by 2.41 fold (± 1.09) at 6 h and 1.43 fold (± 1.07) at 24 h. Overall, insults which resulted in a disruption of the endothelial monolayer corresponded to an increase in MV production. Additionally, the magnitude of the increased production correlated with the degree of hyperpermeability of the endothelial monolayer.

### The GTPase ARF6 is Important in EV (MVs) Biogenesis in BMVECs

**Fig. 3:**
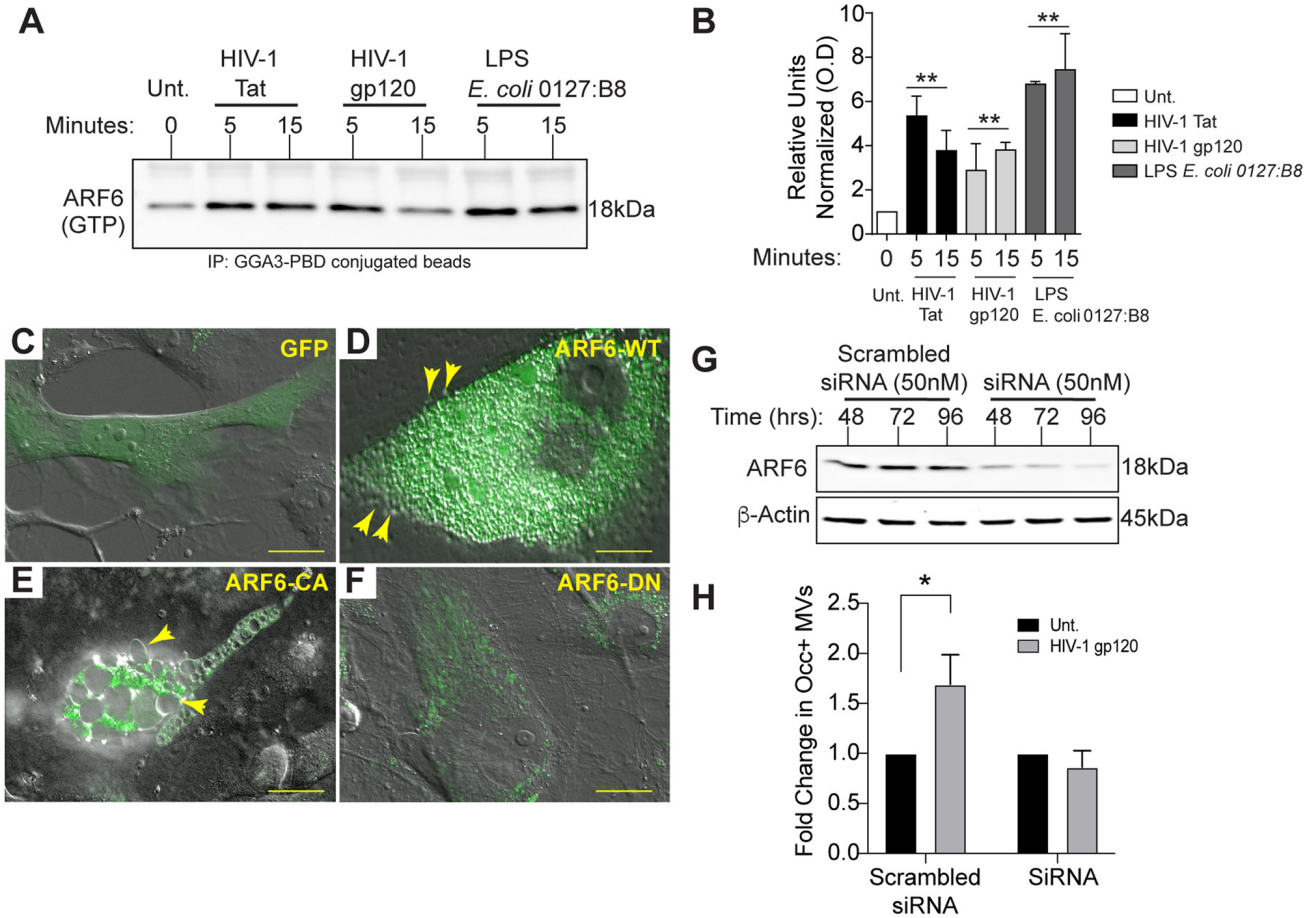
ARF6 is important for MV biogenesis from cerebral vasculature endothelial cells. **A**. Primary human brain microvascular endothelial cells (hBMVECs) were treated with HIV viroproteins Tat and Gp120, or LPS for 5 or 15 min. Activated ARF6 was determined by immunoprecipitation with GGA3 PBD Agarose bead and detection by western blot. **B**. Bar graph quantification of activated ARF6 GTP bands after treatment. Conditions are normalized to the untreated (Unt.) condition. Viral proteins Tat, gp120 as well as LPS statistically increased ARF6 activity at 5 and 15 min after treatment. (n = 4 all conditions, two-tailed t-test to untreated condition. **p < 0.01). C-F. hBMVECs were transfected with ARF6 constructs **C**. pcDNA3-EGFP **D**. pARF6-WT-CFP **E**. pARF6-Q67L-CFP **F** pARF6-T27N-CFP scalebars = 10 microns. Yellow arrow heads indicate budding vesicles and cells were visualized under CFP but were psedocolored for green for better visualization. **G**. ARF6 was knock-down using smart pool siRNA. ARF6 siRNA or scrambled siRNA were transfected into hBMVECs using electroporation. Cell lysates were isolated at 48, 72 and 96 h post-transfection. Representative ARF6 protein levels are shown by western blot. **H**. hBMVECs were treated with siRNA or scrambled siRNA, then treated with gp120 for 6 h. MV were isolated and quantified by flow cytometry. siRNA knock-down of ARF6 prevented the gp120-induced increase in Occludin + MVs

Activation of the small GTP-binding protein, ARF6, has been shown to be important for MV release (Ghossoub et al. 2014; Akers et al. 2013). To examine the role of ARF6 in cerebral endothelial MV biogenesis, hBMVECs were grown and treated with LPS, gp120 and Tat. Activation of ARF6 was determined at 5 and 15 min after treatment using an ARF6 pull-down assay (Fig. 3A). Imaging of nitrocellulose membranes probed for ARF6 showed increased levels of activated ARF6 at 5 and 15 min after treatment with Tat, gp120 and LPS. Quantification of band intensity showed a significant increase of 5.33-fold (± 1.81 SD), 2.88-fold (± 2.43 SD) and 6.77-fold (± 0.26 SD) for Tat, gp120 and LPS, respectively, at 5 min. Furthermore, levels remained significantly elevated through 15 min (Fig. 3B).

To further examine the involvement of ARF6 in MV biogenesis, we transfected hBMVEC with wild type ARF6 and the following mutants fused to YFP: Q67L (constitutively active, Fig. 3E) and T27N (dominant negative, Fig. 3F). Live cell imaging showed that the Q67L mutant had abundant vesicles formed with some showing a budding phenotype. These results suggest that ARF6 activation is induced and that all other cellular machinery for ARF6-mediated MV biogenesis is present in hBMVECs (Fig. 3C–F).

To establish the role of ARF6 in MV generation, siRNA was used to knock-down ARF6 expression. Cells we transfected with siRNA or control scrambled RNA using electroporation and lysates were harvested at 48, 72 and 96 h. Samples were run on SDS-page gels, transferred to nitrocellulose membranes and imaged. ARF6 was significantly reduced with the maximum knock-down in expression at 96 h (Fig. 3H). After establishing the optimal parameters for knock-down, ARF6 was knocked-down and MV production was generated with 100 ng/mL of gp120 for 6 h. In cells transfected with scrambled siRNA, gp120 induced a significant increase in MVs (Fig. 3) similarly to experiments without siRNA (Fig. 2). In cells treated with siRNA, gp120 failed to increase MV production (Fig. 3). Thus, these results support the involvement of ARF6 in MV generation in response to HIV viral proteins.

## Discussion

The discovery that ECs release EVs has generated considerable interest in utilizing EVs as a potential biomarker of vascular health and identification of disease states (Sabatier et al. 2009). In addition, EVs uniquely contain host-specific proteins, which allows for differentiation between EVs from ECs versus platelets and other cell types. Brain endothelium, is unique from other endothelium in that it has highly enriched levels of TJ proteins (TJPs) such as occludin, claudin (3, 5 and 12), zonula occludens protein (ZO 1,2) and junctional adhesion molecules (JAMs) (Abbott et al. 2006). We and others have shown that TJPs are present on vesicles following inflammation and hyperglycemia (Andrews et al. 2016; Paul et al. 2016; Rom et al. 2020). Due to the expression pattern of epithelial cells which also highly expressed TJPs, we hypothesized that the use of a dual marker to identify cerebral vasculature specific MVs can be used to monitor the health of the vasculature and monitor or diagnose cerebral-vasculature damage during HIV infection. Indeed, our data points to the potential of TJP containing MVs as a marker of cerebral dysfunction and correlates with the time kinetics of continued and progressive neuroinflammation resulting from HIV infection that is incompletely resolved with the use of ART.

While our studies are the first to analyze TJ containing EVs from an animal model of HIV infection, several recent studies have examined EVs from HIV patients (Weber et al. 2020; Falasca et al. 2021; Chettimada et al. 2020; Kodidela et al. 2020). Although the isolation methods, vesicle of interest (MV vs. exosome) and target analysis for host origin (monocytes, CNS etc.) vary between studies, several have found increased EVs in people living with HIV (PLWH) (Weber et al. 2020; Chettimada et al. 2020; Kodidela et al. 2020; Hijmans et al. 2019b). With regards to MVs of an endothelial origin, Hijmans et al. found increased endothelial MVs, identified as CD62E + , in HIV + patients on ART compared to healthy individuals (Hijmans et al. 2019b). In contrast, Falasca et al. measured reduced levels of circulating endothelial extracellular MVs (Annexin + /CD31 + /CD41a-CD45-) in HIV + patients on ART compared to healthy individuals (Falasca et al. 2021). While the identification of MVs of an endothelial origin varied between studies, Falasca et al. also did not perform an EV isolation method (ultracentrifugation, filtration etc.) and instead stained for MVs in whole blood samples. Overall, further studies are needed in order to determine if the results of our animal model of HIV infection translate to PLWH.

Our data demonstrates that primary human brain endothelial cells release MVs containing TJPs in response to HIV proteins and inflammatory cytokines. This response of elevated occludin containing MV production could potentially extend to any insult resulting in a disassembly of TJPs. The production of EVs from various cell types have been characterized to occur during HIV pathogenesis. However, not much is known about the EV response from brain endothelial cells during the course of viral infection. The only report that points to this possibility notes that EVs are released by cultured BMVEC from the human-derived cell line, hCMEC/D3, when these cells are exposed to HIV viral particles (Andras et al. 2017). Although particle concentration was analyzed, the cargo from these cells was not. Our Fig. 2 corroborates their findings, showing generation of EVs following exposure of primary human BMVECs to recombinant HIV proteins. Furthermore, these results suggest that BMVECs are sensitive to HIV-1 viral proteins and respond by generating EVs that contain proteins associated with the TJP complex. Additionally, human ortic ECs have also been reported to produce elevated MVs (positive for CD144, VE-Cadherin) in response to gp120 and Tat (Hijmans et al. 2019a) which indicates that all ECs may have a similar elevated MV response to HIV viroproteins.

While there is potential to utilize EVs as biomarkers of vasculature health, the significance of these vesicles on pathology remains unclear. We and others have found evidence that vesicles aid in the transmigration of leukocytes into the CNS (Ramirez et al. 2018; Paul et al. 2016). Thus, there is also the potential to mitigate the progression of disease pathology through mechanisms regulating the production of these EVs. For instance, key questions remain as to whether prevention of these vesicles alone can slow or reverse the deleterious vascular pathology or immune infiltration during neuroinflammation. Thus, we sought to define key molecules regulating vesicle production in response to the HIV viral protein gp120. Our results using ARF6 activation assays and siRNA knockdown demonstrate the importance of ARF6 activation in the release of increased quantities of vesicles. Future studies to investigate whether manipulation of these pathways can alter the disease progression could open the door to new therapeutic interventional strategies.

## Summary

HIV Infection is well established to have negative consequences on the cerebral vasculature which persists even with viral suppression using ART (Marincowitz et al. 2019). We demonstrate the detection of elevated quantities of brain-EC derived MVs after HIV infection and was attenuated by ART. Furthermore, we show that MV release from hBMVECs follows the disruption of barrier integrity. MV biogenesis in response to the HIV viroprotein gp120 was mediated by ARF6. Finally, EV release may reflect active remodeling of the brain vasculature and serve as a biomarker readout for BBB status for a broad range of CNS neuropathies including HAND.
